# Information-Motivation-Behavioral Skills model of consistent condom use among transgender women in Shenyang, China

**DOI:** 10.1186/s12889-020-08494-y

**Published:** 2020-03-26

**Authors:** Huwen Wang, Ruijie Chang, Qiuming Shen, Lhakpa Tsamlag, Shuxian Zhang, Yue Shi, Tiecheng Ma, Zixin Wang, Rui She, Joseph T. F. Lau, Ying Wang, Yong Cai

**Affiliations:** 1 School of Public Health, School of Medicine, Shanghai Jiao Tong University, No.227, South Chongqing Road, Shanghai, 200025 People’s Republic of China; 2 China Love Aid, Shenyang, 110000 People’s Republic of China; 3 Centre for Health Behaviors Research, JC School of Public Health and Primary Care, the Chinese University of Hong Kong, Hong Kong, SAR China

**Keywords:** Transgender women, Condom use, Information-motivation-behavioral skills model, HIV/AIDS, China

## Abstract

**Background:**

The Information-Motivation-Behavioral Skills (IMB) model has received consistent empirical support in the context of HIV prevention among various key populations, but not yet among transgender women (TGW). None effective interventions has been carried out among TGW so far to control their high prevalence of unprotected anal intercourse. The intent of the current study is to examine the application of the IMB model to clarifying the association between condom use correlates and condom use frequency among TGW in China.

**Methods:**

Using snowball sampling, we recruited 198 self-identified TGW in Shenyang, China from April 2017 to July 2017. Participants were required to complete a questionnaire assessing their background characteristics and IMB model constructs. Structural equation modeling (SEM) was conducted to demonstrate the utility of the IMB model.

**Results:**

The consistent condom use (CCU) rate was 47.0%. Results of SEM indicated that HIV-preventive motivation (comprising condom use attitude and subjective norms; β = 0.823, *P* <  0.001) and behavioral skills (including condom use skills and self-efficacy; β = 0.979, *P* = 0.004) were related to more frequent condom use, whereas HIV knowledge was unrelated to condom use (β = 0.052, *P* = 0.540).

**Conclusions:**

The low CCU rate suggested that TGW in China were at high risk of HIV infection and transmission and a key intervention population. HIV-preventive interventions for this population should focus on enhancing motivation and strengthening behavioral skills to increase condom use frequency and reduce HIV infection.

## Background

Transgender women (TGW) are those whose gender identity (woman) is different from their birth-assigned sex (male) [1]. Scarce as this population is (0.5–0.9% of the whole population are identified as transgender worldwide [2–4]), their HIV prevalence is remarkable. The overall HIV prevalence (self-reported and laboratory confirmed) for TGW worldwide is 18.8–28% [1, 5–8]; this group has a 49-fold higher odds of infection compared with other reproductive age adults [6], and twice the odds compared with men who have sex with men (MSM) [5]. In China, TGW are not covered by routine HIV surveillance [9], but several small-sample studies suggest TGW’s high HIV prevalence rate. The number might even be underestimated because of low rates of HIV testing among this particular population in China [9]. For example, in a cross-sectional study among 220 TGW sex workers conducted in 2014, 16.8% of the participants self-reported as HIV positive and another 9.1% were detected to be HIV positive through free HIV testing [10].

Among all risky sexual behaviors, unprotected anal intercourse (UAI; anal intercourse without condoms) has the highest risk for HIV acquisition and transmission among TGW [11, 12]. Pinkerton et al. found that inconsistent or non-condom users were 10–20 times more likely to acquire HIV than consistent condom users [13]. Promoting consistent condom use among key population has been verified to be a promising risk reduction strategy. Smith et al. estimated that condom effectiveness of reducing HIV incidence was 72.3% (95% confidence intervals [CI]: 60.7–80.5%) for receptive anal sex, 62.9% (95% CI: 46.3–74.3%) for insertive anal sex, and 70.5% (95% CI: 58.2–79.2%) for any receptive or insertive anal sex [12]. A meta-analysis by the World Health Organization of five cohort studies [14] assessed condom effectiveness among MSM and transgender people in developed countries and found that the relative effect on HIV transmission of condom use was a relative risk of 0.36 (95% CI: 0.20–0.67) and that consistent condom use (CCU) reduced HIV transmission by 64% (95% CI: 32–80%).

However, none effective interventions have been carried out among TGW so far. UAI prevalence remains high among TGW, even higher than other sex/gender minorities. A cross-sectional study conducted by Shan et al. in two Chinese provinces found that 81.5% TGW reported not always using condoms with stable partners, and 70.9% not with casual partners (Shan et al., 2018). According to Poteat et al., TGW had more than twice the odds as MSM of reporting condomless receptive anal sex with male partners (Poteat et al., 2017). The high prevalence of UAI highlights the urgent need for targeted and effective prevention interventions targeting this population.

Theory-based intervention is widely believed to be more likely to effectively change behavior and maintain such behavior change than non-theory-based intervention. Among extant theory and models, the Information-Motivation-Behavioral Skills (IMB) model has received consistent empirical support in the context of HIV prevention among various key populations, including heterosexual people living with HIV [15], sexually transmitted infection (STI) clinic patients [16], female sex workers [17], senior high school students [18, 19], college students [20], male street laborers [21], unmarried female migrants [22], and MSM [23].

The model posits that HIV-preventive behavior is a function of individuals’ knowledge (information) of HIV transmission and prevention, their motivation to carry out HIV prevention (attitudes toward and subjective norms regarding HIV-preventive acts), and their skills in conducting specific preventive behaviors (possession of the requisite skills and a sense of self-efficacy) [24, 25]. The model further assumes that information and motivation may have direct (when complicated or novel behavioral skills are unnecessary to behavior) or indirect effects (through behavioral skills) on behavior [24, 25].

Fisher et al. pointed out that the IMB model constructs were highly generalizable determinants of HIV-preventive behavior in diverse populations of interest, but these constructs should have content that is specific to particular target populations as well as to particular HIV-preventive behaviors [24]. To the best of our knowledge, there have been no tests of the utility of the IMB model of condom use among TGW. It is therefore important to conduct a test before using it to guide the development of interventions targeting this population.

Based on the above-mentioned evidence, we intended to examine the application of the IMB model to clarifying the association between condom use correlates and condom use frequency among TGW in China. Theoretical evidence was expected to be provided to guide the development of interventions targeting increasing condom use frequency in this population. We hypothesized that information and motivation would affect condom use directly and indirectly through behavioral skills and would be independent from each other.

## Methods

### Study setting

Subjects were recruited in Shenyang (provincial capital of Liaoning Province), China between April 2017 and July 2017. According to the sixth national population census of the People’s Republic of China [26], Shenyang is the largest city in Northeast China by urban population, with 8.1 million resident population. The number of MSM population in Shenyang ranks the second in China with an estimated 140,000 MSM in 2006 [27]. MSM population accounted for 81.2% of the city’s annual newly reported HIV cases [28], while the national average proportion is only 28.3% [29].

### Participants

The inclusion criterion were: 1) older than 18 years; 2) male birth assigned sex; 3) self-identified as woman; 4) reporting having sexual intercourse experiences in the last 6 months; 5) agreeing to participate in the study and willing to provide written informed consent.

### Recruitment

Snowball sampling [30, 31] was used for recruitment because TGW in China are hard to reach and hidden due to the universal discrimination [32]. And in the absence of a sampling frame, it was not feasible to perform probability sampling. The participants were recruited through a non-governmental organization (NGO), China Love Aid, which dedicated to improving the physiological and psychological health of TGW. With the assistance of the NGO, five participants were recruited as the ‘seeds’. The five seeds then each recruited or referred other participants, thus, aggregating the snowball until saturation reached. The saturation was also double-checked by the NGO leaders, who worked with local TGW and knew the approximate number of TGW living locally. All eligible participants were asked to read and sign informed consent documents for voluntary participation after being informed of the following: all data collected were anonymous and strictly confidential and would be used only for research; refusal would not affect their right to use any service; they could quit at any time without being questioned.

### Data collection

A total of 198 eligible TGW provided written informed consent and participated in this study. They were then asked to complete the questionnaires during a face-to-face interview in a private room by trained investigators, who were staff of the NGO, to ensure the quality and completeness of the response. The questionnaires were anonymous and took approximately 30 min to complete. All subjects were paid 200 CNY (about 30 USD) as remuneration after completion of the above procedure. The ethics committee of the School of Public Health, Shanghai Jiao Tong University, approved the recruitment procedure and the study protocol.

### Measures

#### Background characteristics

The following background characteristics were collected, including age, educational level, marital status, monthly income, and length of residence in Shenyang.

#### Information

To assess knowledge deficits and misconceptions about HIV risks and self-protective behaviors, the 18-item true–false Brief HIV Knowledge Questionnaire [33] (Cronbach’s α = 0.683) was administered. The HIV knowledge score was obtained by summing the number of correct responses. Higher scores indicated greater information.

#### Motivation

Motivation to engage in condom use was measured in accord with the principal constructs of the Theory of Reasoned Action [34] and the Health Belief Model [35]. Two scales were developed by the authors to provide two separate indicators of motivation. A total score was obtained by summing scores for individual items. Higher scores on the two scales indicated stronger motivation.

The 6-item, Likert type Attitude Scale (Cronbach’s α = 0.528) assessed subjects’ attitudes toward condom use. For favorable statements (“I think condoms can prevent the transmission of HIV”; “I think condoms should be used during anal intercourse”; “I will try to persuade my partner into using condoms”), the strongly agree response is given a weight of 5 and the strongly disagree response a weight of 1, with other responses weighted accordingly. For unfavorable statements (“I think using condoms during anal intercourse is uncomfortable for both partners”; “I think condoms are unreliable in preventing HIV”; “In my opinion, condoms are too much trouble”), the scoring is reversed.

The 4-item Subjective Norms Scale (Cronbach’s α = 0.806) assessed perceptions of what others (family members, friends, peers who are engaged in the same work, and significant others) think about the participants’ consistent condom use during anal sexual intercourse, where 1 = strongly disapprove, and 5 = strongly approve.

#### Behavioral skills

To assess subjects’ HIV-preventive behavioral skills, the 6-item Condom Influence Strategy Questionnaire (CISQ-S) [36] (Cronbach’s α = 0.873) and the 6-item Self-Efficacy Scale (Cronbach’s α = 0.874) developed by the authors were administered. Each scale represented a separate indicator of behavioral skills. Higher scores on both scales indicated better behavioral skills.

The CISQ-S assessed subjects’ condom use negotiation skills and unprotected sex refusal skills. Questions were answered in a Likert format that ranged from 1 = never to 5 = always. An example item from the scale is “I would like to discuss condom use with my partner”.

The Self-Efficacy Scale assessed their ability to engage in condom use effectively by these questions: “I can carry a condom with me in case I need it”; “I can use a new condom every time I have anal sex”; “I can use condoms throughout my anal sex”; “I can avoid the slipping of the condom during anal sex”; “I can discuss condom use with my partner even after drinking alcohol”; “I can negotiate using condoms with my partner no matter who he is”. Participants answered on a 5-point Likert scale that ranged from 1 = definitely can’t do to 5 definitely can do.

#### Condom use

The questionnaire contained three frequency rating items (all scaled from 1 = never to 4 = always) to assess subjects’ condom use with their regular sexual partners (defined as those who were in a stable relationship such as boyfriends that did not involve transactional sex), casual sexual partners (defined as those who were not regular partners where transactional sex was not involved), and transactional sexual partners (defined as those with whom transactional sex was involved) during the last 6 months. Similar definition has been widely used in other studies (e.g. [37]). To compare background characteristics between subjects with and without CCU, a dichotomous variable were created, with 1 indicating always using condom with all types of sexual partners, and 0 indicating not always using condom with at least one type of partners. Besides, a total condom use frequency was obtained by calculating the average frequency of use with the three types of sexual partners. Higher scores indicated more frequent condom use.

### Statistical analysis

Descriptive analyses were conducted using IBM SPSS Statistics 23.0 (IBM Corp., Armonk, NY, USA). Comparisons were conducted between subjects showing CCU and those not showing CCU to detect potential risk factors using Student’s t-test and Pearson’s chi-square test. Spearman’s correlation analysis was conducted to examine correlations among IMB model constructs. Confirmatory factor analysis (CFA) and structural equation modeling (SEM) were conducted using IBM SPSS AMOS 21.0 (IBM Corp., Chicago, IL, USA). The CFA measurement model included two latent constructs (motivation, behavioral skills) that predicted its indicators (motivation: condom use attitude, subjective norms; behavioral skills: condom use skills, self-efficacy). Once an acceptable measurement model had been established, a structural equation model was built to examine correlates of condom use among TGW based on the IMB model. Model fit was assessed using the ratio of chi-square values to degrees of freedom (χ^2^/df), the comparative fit index (CFI), and the root mean square error of approximation (RMSEA) [38]. A χ^2^/df ratio of 3 or less, a CFI greater than 0.90, and an RMSEA lower than 0.08 indicated acceptable model fit [38, 39].

## Results

### Background characteristics of subjects

A total of 198 TGW completed the questionnaire (Table 1). They had an average age of 33.5 years (standard deviation = 9.6; range: 18–62 years). Most participants had a junior high school education or above (92.4%), were single (77.3%), and had a monthly income of 3001–6000 CNY (48.5%). Only 35.4% were local residents, with the rest 64.6% migrating to Shenyang. Nearly half the subjects (47.0%) reported CCU; the CCU rates with regular sexual partners, casual sexual partners, and transactional sexual partners were 26.3, 28.8, and 59.1%, respectively. CCU subjects were significantly younger (31.9 vs. 34.9 years; *P* = 0.030) and tended to be better educated than non-CCU subjects (62.3% vs. 40.0% with senior high school level or above; *P* = 0.005).

**Table 1:** Background characteristics of all subjects and comparisons between CCU and non-CCU subjects

Characteristics	All subjects (*N* = 198) (n[%])	Subjects with CCU (*n* = 93) (n[%])	Subjects without CCU (*n* = 105) (n[%])	*P* value
Age (years) (mean ± SD) (range)	33.5 ± 9.6 (18–62)	31.9 ± 9.4 (18–59)	34.9 ± 9.6 (18–62)	**0.030**
Educational level				**0.005**
Primary school and below	15 (7.6)	8 (8.6)	7 (6.7)	
Junior high school	83 (41.9)	27 (29.0)	56 (53.3)	
Senior high school	50 (25.3)	27 (29.0)	23 (21.9)	
College and above	50 (25.3)	31 (33.3)	19 (18.1)	
Marital status				0.164
Single	153 (77.3)	77 (82.8)	76 (72.4)	
Married	11 (5.6)	5 (5.4)	6 (5.7)	
Divorced	34 (17.2)	11 (11.8)	23 (21.9)	
Monthly income (CNY)				0.525
≤ 3000	46 (23.2)	23 (24.7)	23 (21.9)	
3001–6000	96 (48.5)	40 (43.0)	56 (53.3)	
6001–12,000	41 (20.7)	22 (23.7)	19 (18.1)	
> 12,000	15 (7.6)	8 (8.6)	7 (6.7)	
Length of residence in Shenyang				0.237
Local residents	70 (35.4)	31 (33.3)	39 (37.1)	
Living locally ≤1 year	19 (9.6)	13 (14.0)	6 (5.7)	
Living locally 1–5 years	50 (25.3)	24 (25.8)	26 (24.8)	
Living locally > 5 years	59 (29.8)	25 (26.9)	34 (32.4)	
CCU	93 (47.0)			
With regular sexual partners (*n* = 115)	52 (26.3)	–	–	–
With casual sexual partners (*n* = 117)	57 (28.8)	–	–	–
With transactional sexual partners (*n* = 170)	117 (59.1)	–	–	–

*CCU* consistent condom use, *SD* standard deviation

*P* value < 0.05 was considered significant (in bold)

### Bivariate associations between variables

The bivariate correlations among the IMB model constructs are shown in Table 2. HIV knowledge did not correlate with any model variables (attitude toward condom use: *r* = 0.124, *P* = 0.083; subjective norms: *r* = 0.040, *P* = 0.571; condom use skills: *r* = 0.038, *P* = 0.594; self-efficacy: *r* = 0.027, *P* = 0.708; condom use frequency: *r* = − 0.030, *P* = 0.670). Condom use frequency was associated with all variables except HIV knowledge and subjective norms (*r* = 0.030, *P* = 0.673). Apart from the aforementioned correlations, all the variables were significantly associated with the other ones.

**Table 2:** Spearman’s correlation analysis of IMB model constructs

IMB variables						
HIV knowledge	1.000					
Attitude toward condom use	0.124	1.000				
Subjective norms	0.040	0.291***	1.000			
Condom use skills	0.038	0.330***	0.288***	1.000		
Self-efficacy	0.027	0.365***	0.312***	0.683***	1.000	
Condom use frequency	−0.030	0.228**	0.030	0.491***	0.442***	1.000

***P < 0.001, **P < 0.05. *IMB* Information-Motivation-Behavioral Skills

### Confirmatory factor analysis

The model fit indices indicated an acceptable model fit: χ^2^ = 0.046, df = 1, χ^2^/df ratio = 0.046 < 3, CFI = 1.000 > 0.90, and RMSEA = 0.000 <  0.08. Given the overall fit of the data to the model, no additional paths or co-variances were added.

Table 3 shows the ranges, medians, interquartile ranges, and factor loadings for the IMB model constructs. The median percentage of correct responses to HIV knowledge was 61.1% (11.0/18). The median scores for attitude toward condom use, subjective norms, condom use skills, self-efficacy, and condom use frequency was 23.0/30 (76.7%), 16.0/20 (80.0%), 26.0/30 (86.7%), 28.0/30 (93.3%), 3.5/4 (87.5%), respectively. All factor loadings in the model were significant (*P* < 0.001), indicating that the hypothesized latent construct predicted its proposed manifest indicators well.

**Table 3:** Ranges, medians, interquartile ranges, and factor loadings for the IMB model constructs

Items	Range	Median (25th–75th quartile)	FL
Information			
HIV knowledge	0–18	11.0 (9.0–13.0)	–
Motivation			
Attitude toward condom use	6–30	23.0 (22.0–26.0)	0.567
Subjective norms	4–20	16.0 (14.0–18.0)	0.542
Behavioral skills			
Condom use skills	6–30	26.0 (21.0–29.3)	0.872
Self-efficacy	6–30	28.0 (24.8–30.0)	0.883
Behavior			
Condom use frequency	1–4	3.5 (3.0–4.0)	–

*IMB* Information-Motivation-Behavioral Skills, *FL* factor loading, all significant at *P* < 0.001

### The IMB model

**Fig. 1:**
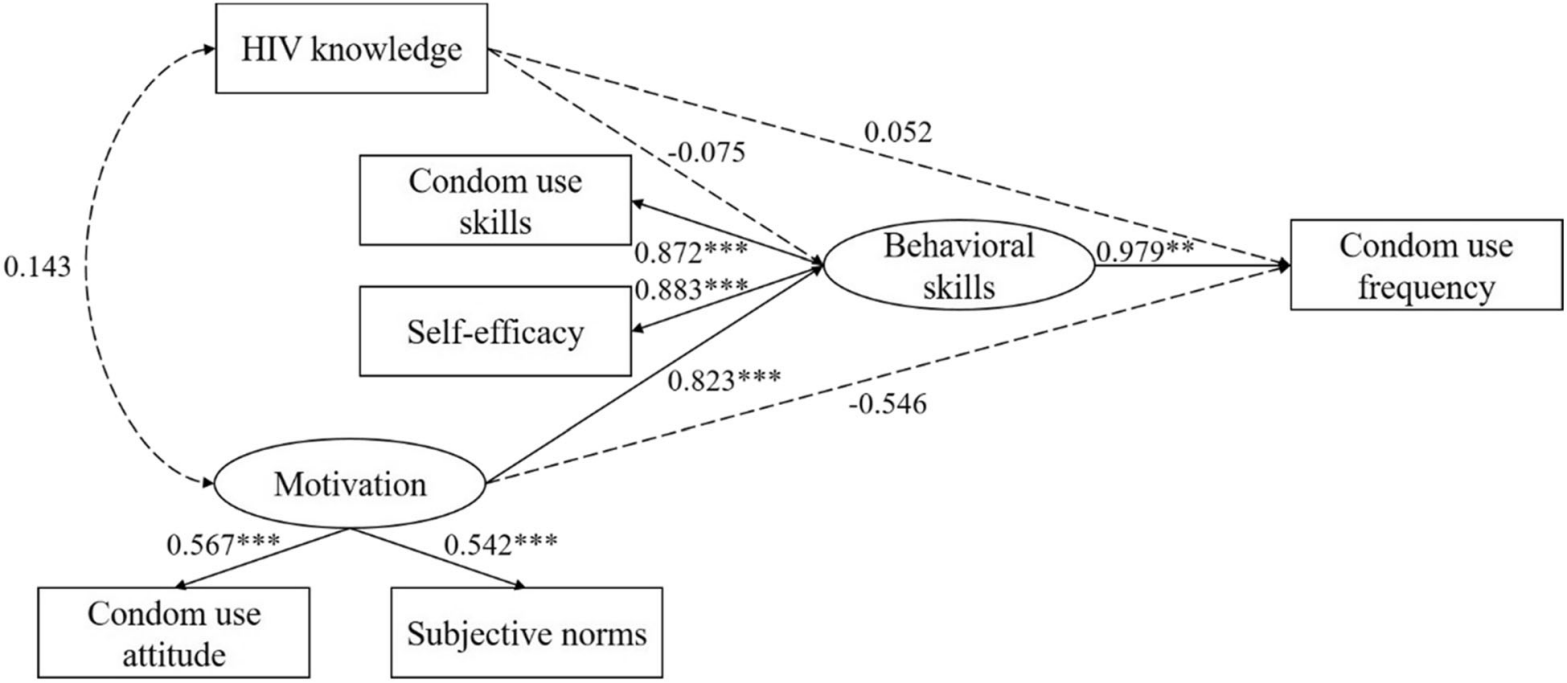
The Information-Motivation-Behavioral Skills model of condom use frequency among transgender women in China (*N* = 198). Path coefficients are standardized. Circles represent latent variables and rectangles represent manifest variables. Single arrows represent standardized regression coefficients and multiheaded arrows represent standardized correlations. Solid lines represent statistical significance and dashed lines represent a lack of statistical significance. **P < 0.01, ***P < 0.001

The model was depicted in Fig. 1. The model fit indices indicated an acceptable model fit: χ^2^ = 11.258, df = 5, χ^2^/df ratio = 2.252 < 3, CFI = 0.980 > 0.90, and RMSEA = 0.080. Condom use frequency was strongly associated with behavioral skills (β = 0.979, *P* = 0.004). Motivation had no direct effect on condom use frequency but had a reliable effect on behavioral skills (β = 0.823, *P* < 0.001), which in turn significantly affected condom use frequency. Contrary to the IMB model, knowledge was not associated with either behavioral skills (β = − 0.075, *P* = 0.372) or condom use frequency (β = 0.052, *P* = 0.540) and neither was it a covariate of motivation (*r* = 0.143, *P* = 0.172). In combination, these variables accounted for 38.7% of the variation in condom use frequency. Path coefficients were showed in detail in Table 4.

**Table 4:** Path coefficients of the IMB model

Path	Estimate	Std. Estimate	S.E.	*P* value
Information↔Motivation	0.727	0.143	0.532	0.172
Information→Behavioral skills	−0.115	−0.075	0.129	0.372
Information→Condom use frequency	0.013	0.052	0.021	0.540
Motivation→Behavioral skills	2.477	0.823	0.577	**< 0.001**
Motivation→Condom use frequency	−0.266	−0.546	0.186	0.152
Behavioral skills→Condom use frequency	0.158	0.979	0.054	**0.004**

*Std. Estimate* standardized estimate, *S.E*. standard error

*P* value < 0.05 was considered significant (in bold)

## Discussion

The results of this study demonstrated the utility of the IMB model for understanding condom use among TGW in China. The IMB model fit the data well and accounted for 38.7% of the variance in condom use frequency. The findings supported some, but not all, of the hypothesized associations between information, motivation, behavioral skills and condom use.

Results showed that the CCU rate (47.0%) was much higher than that reported in previous Chinese research (18.5% with stable partners, 29.1% with casual partners, 16.7% when buying sex, and 14.0 when selling sex) [40]. We attributed it to the higher proportions of subjects reporting transactional sexual intercourse in the current sample compared to the previous study; 170/198 (85.9%) have had transactional sexual intercourse in the previous 6 months. Cambou et al. discovered that unprotected anal intercourse was more prevalent in partnerships with a greater degree of interpersonal commitment like stable partners [41]. So condom use with transactional sexual partners was normally more frequent than stable and casual partners, which buffered the overall rate. Variable CCU rates with different types of sexual partners detected in the current study proved this speculation; CCU rates with regular sexual partners, casual sexual partners, and transactional sexual partners were 26.3, 28.8, and 59.1%, respectively. Anyway, the overall CCU rate in China was much lower than that reported in previous systematic research among US transgender population (61.8%) [1], indicating the poor control of high risk sexual behavior among Chinese TGW and appealing to urgent and effective intervention.

Furtherly, a SEM was conducted to demonstrate the utility of the IMB model in clarifying the association among information, motivation, behavioral skills and condom use. Inconsistent with our hypothesis, motivation was not directly associated with condom use. However, in accord with some previous studies [19, 42], the effect of motivation on condom use was completely mediated by behavioral skills. More specifically, TGW who are highly motivated (who have a positive attitude and strong subjective norms about condom use) are likely to acquire the requisite skills (condom use skills and self-efficacy) for condom use. The results highlighted the crucial role of behavioral skills in promoting condom use.

While condom use is the most reliable method to reduce HIV risk, it is also a complex sexual behavior. Previous studies found that using a condom conveys potentially undesirable implicit messages such as lacking trust, lacking commitment or being HIV/STI-infected [43]. As such, complicated skills, such as condom use negotiation, were needed to convert one’s safer-sex intentions into practical safer-sex behaviors (e.g. condom use). The important role of behavioral skills on condom use has been proved among other populations in previous studies. A meta-analysis of psychosocial correlates associated with heterosexual condom use found that partner communication about condom use was the most highly correlated factor in association with condom use [44]. The intervention effect of behavioral skills has also been verified among other populations. Gause et al. concluded through their meta-analysis that participants who were exposed to HIV prevention interventions targeting improved partner communication used condoms more frequently [43]. It’s reasonable to speculate based on our findings and present studies that intervention targeting improving TGW’s condom use behavioral skills can significantly elevate frequency of condom use among this population.

Consistent with the IMB model, correlation between motivation and information was insignificant, identifying motivation and information as independent constructs [11]. The path from motivation to condom use by way of behavioral skills can exist independently. It indicated that well-informed was an unnecessary prerequisite for an individual to be well-motivated.

Contrary to the IMB model, information showed no association with behavioral skills and condom use. Previous tests of the IMB model have found inconsistencies in the relationship between information and behavioral skills [19, 21, 22] and between information and condom use [16–18, 20, 23, 24]. Some researchers ascribed it to the speculation that information might impact initial behavioral change rather than maintain such behavior over time [22, 45]. Others speculated that one’s information might have limited impact on dyadic behavioral change such as condom use, which involves communication and negotiation with his partner [22, 46]. In the current study, the lack of association between information and behavioral skills may be further ascribed to these two constructs not being measured at the same level of specificity [16]; information comprised knowledge about HIV transmission and self-protective behaviors, while behavioral skills consisted of condom use negotiation skills and unprotected sex refusal skills. Nevertheless, results of information provided some hints for future intervention. Subjects showed obvious deficits and misconceptions about HIV risks and self-protective behaviors (11.0/18) in the current study, indicating that the extant health propaganda and education on HIV prevention in China remains insufficient among TGW population.

The present study has some limitations that should be acknowledged. Firstly, cross-sectional study limits our ability to make causal inference. Longitudinal studies are needed to further verify the causal mechanism. Secondly, for lack of sampling frame, the participants were recruited by non-probability sampling approaches, namely snowball sampling. Sampling bias might exist. Another method for recruitment of hard to reach population, which is widely believed to be better than snowball sampling, is respondent-driven sampling (RDS). RDS lends statistical rigor to conventional snowball sampling through longer recruitment chains, recruitment limits, and the collection of data used to statistically adjust for the biases inherent in how persons of similar characteristics are networked and likely to recruit each other. Nevertheless, RDS also needs much higher funds than snowball sampling because with each qualified new participant recruited by the seeds in RDS, extra financial reimbursements are needed. Limited to research grant, we were only able to provide such a high reimbursement (200 CNY, about 30 USD) to the participants for completing the questionnaires alone (i.e. no more money for referral), in order to guarantee the quality of the answers provided by the participants. In future research, we will figure out a better method to solve this problem. Furthermore, as the subjects were all from Shenyang, Liaoning Province, it may not be possible to generalize the findings to TGW from other locations. Lastly, some IMB model studies among other populations have used it to compare different groups (such as patients reporting prior STI treatment versus those not reporting such treatment (Scott-Sheldon et al., 2010)) to define a more specific target subgroup. The CCU rate showed substantial variation across different types of sexual partners in this study, indicating possible disparity of the modeling results. Limited to sample size, we failed to make further explorations. Future research is recommended to make clear the mechanisms underlying how information, motivation, and behavioral skills affect condom use with different types of sexual partners among TGW.

## Conclusions

The current study is the first to examine the determinants of condom use frequency in TGW using the IMB model. Findings indicated that interventions for this population should focus simultaneously on enhancing motivation and strengthening behavioral skills regarding condom use to substantially increase HIV-preventive behavior. Besides, although information was not significant in the model, participants’ scores on information indicated that health propaganda and education on HIV prevention among TGW population in China are far from sufficient and need further improvements.

## Data Availability

The datasets used during the current study are available from the corresponding author on reasonable request.

## Abbreviations

CCU: Consistent condom use
CFA: Confirmatory factor analysis
CFI: Comparative fit index
CI: Confidence intervals
CISQ-S: Condom Influence Strategy Questionnaire
FL: Factor loading
IMB: Information-Motivation-Behavioral Skills
MSM: Men who have sex with men
NGO: Non-governmental organization
RMSEA: Root mean square error of approximation
S.E.: Standard error
SEM: Structural equation modeling
Std. Estimate: Standardized estimate
STI: Sexually transmitted infection
TGW: Transgender women
UAI: Unprotected anal intercourse
χ^2^/df: Chi-square values to degrees of freedom
